# Treatment of Diseases of the Testicle by Compression

**Published:** 1841-02

**Authors:** 


*Treatment of Diseases of the Testicle by Compression.*—Langston Parker, Esq., extols this practice as possessing great advantages over the ordinary methods. "In common swelled testicle compression may be employed," he says, "as soon as the patient is able to bear the application of the plasters; and this is generally much sooner than might be supposed, however acute the disease may be. The great advantages possessed by compression are, almost immediate relief to pain; which is occasioned, in great measure, by the dragging and weight of the inflamed organ, and is mitigated, and most common.y completely removed by its support: the patient can pursue, in most instances, his ordinary duties, and there is no occasion to confine him to bed; a circumstance of great importance where concealment is wished, as it always is, in diseases of this kind. In addition the cure is much more quickly, and much more perfectly affected by compression than under the ordinary method, the swelling of the gland more rapidiy subsiding, and less commonly leaving behind it any chronic irritation and enlargement, which is liable at some future time, in bad constitutions, from injury, or from a renewal of the disease, to degenerate into some of the malignant forms of disease of the testis.

"Chronic inflammation and enlargement of the testis also very commonly occurs as one of the symptoms of constitutional syphilis, as well as from other causes. Here, also, compression, as a local remedy, is by far the most valuable of any with which I am acquainted, particularly in those cases where enlargement of the organ, from chronic inflammation, has taken place to a considerable extent, where the weight chiefly incommodes the patient, and little or no tenderness is experienced when the part is handled.

"I am not prepared to say how far compression may be of service in the incipient stages of certain forms of malignant disease of the testis, but believe much advantage may be derived from its employment, since, in addition to the benefit to be looked for from mere compression of the part, much good may be expected from the constant topical application of mercury, iodine, and other remedies, only to be well accomplished in this way.

"Compression of the testis is to be practised by surrounding the organ by straps of plaster, applied as closely and as tight as the patient can bear. The plasters I generally use are those of ammoniacum, with mercury, or iodine and belladonna. These are to be smoothly spread upon thick wash leather, and cut into thin straps; an assistant then grasps the tisticle, and draws it as far downwards as he can in the scrotum, which should previously be washed with a little spirits of wine. The first strap should be placed at the upper part, circularly round the testis, and succeeded by others, placed in the same direclion, till the whole is covered. A second series of straps is then to be placed in an opposite direction to the former, crossing them at right angles, from behind forwards. One or two long ones may then be placed over these, where they appear to be most needed, to keep the whole in place. The parts should be supported by a well-fitting suspensory bandage, although the plasters at once relieve any inconvenience or pain that may have been occasioned by the dragging or weight of the testicle."—*Lancet*, July 25, 1840. —*Ibid.*

